# Fermented rye with *Agaricus subrufescens* and mannan-rich hydrolysate based feed additive to modulate post-weaning piglet immune response

**DOI:** 10.1186/s40813-021-00241-y

**Published:** 2021-12-09

**Authors:** Nienke de Groot, Fernando Fariñas, Lluís Fabà, Ellen Hambrecht, Carolina G. Cabrera-Gómez, Francisco J. Pallares, Guillermo Ramis

**Affiliations:** 1 Trouw Nutrition Innovation, Amersfoort, 3811 MH The Netherlands; 2 Instituto de Inmunología Clínica y Enfermedades Infecciosas, Málaga, Spain; 3 Dpto. Producción Animal, Facultad de Veterinaria, Universidad de Murcia, Murcia, Spain; 4 Dpto. Anatomía y Anatomía Patológica Comparadas, Universidad de Murcia, Murcia, Spain

**Keywords:** Inflammation, Cytokines, Additive, Intestine, Piglets, Weaning

## Abstract

**Background:**

The process of weaning in piglets is often associated with an increased inflammation response in the intestine and compromised intestinal integrity and morphology, favoring a delay in intestinal maturation and a predisposal to diseases. Research has shown the potential of different nutritional strategies to reduce the production of pro-inflammatory cytokines, with the main goal to manipulate health and performance of pigs. Promising examples of nutritional strategies are fungal fermented products and their derivatives which are described to contain several compounds that may play a role in gastrointestinal health and pathogenic bacteria control. Products from *Agaricus subrufescens* mushroom are reported to contain prophylactic and therapeutic properties including antimicrobial and immunomodulatory properties.

**Results:**

This study analysed the post-weaning immune status in intestinal tissue and blood of piglets, with the objective to evaluate the gastrointestinal health and immune modulation response induced by a blend of mannan-rich hydrolyzed copra meal and fermented rye with *A. subrufescens*. Intestinal histomorphology demonstrated a villus height reduction in jejunum and increase in ileum on day 15, while increased villous height in jejunum and ileum on day 30. The results showed that in post-weaning piglets, the feed additive stimulates an immunomodulation effect most evident at 15 days post-weaning, with significant lower expression of cytokines Interferon (IFN) γ, Interleukin (IL) 1α, IL-1β, IL-6, IL-8, IL-10 and Transforming Growth Factor (TGF) β in jejunum, accompanied with an increase in peripheral blood mononuclear cells (PBMC) cytokine gene expression of IL-1β, IL-6, IL-8, IL-10, IL-12p35 (IL-12α), IL-12p40 (IL-12β), Tumor Necrosis Factor (TNF) α, IFN-α, and TGF-β. In piglets fed the feed additive, the quantity of Immunoglobulin (Ig) A producing cells in jejunum, ileum was reduced on day 15 and 30 post-weaning, and on day 30 and 45 post-weaning in colon tissue. Natural Killer (NK) cells count in blood were increased on day 15 post-weaning in the piglets fed the feed additive.

**Conclusion:**

This study implies the potential of the blend including mannan-rich hydrolyzed copra meal and fermented rye with *A. subrufescens* on immune modulation in the intestine of post-weaning piglets.

**Supplementary Information:**

The online version contains supplementary material available at 10.1186/s40813-021-00241-y.

## Background

The gastrointestinal tract (GIT) is essential in the maintenance of health. The gut mucosal immune system alone contains more than 10^12^ lymphocytes and has a greater concentration of antibodies than other tissues in the body [1]. The intestine main function is the uptake of nutrients while simultaneously form a physical barrier which should prevent toxic compounds and pathogens from crossing the intestinal mucosa and systemic circulation. Two major components of the intestinal barrier are the intestinal epithelium and the gut associated lymphoid tissue (GALT). This tissue has the challenging dual task of selectively absorbing nutrients from the intestinal lumen, while preventing microbial and toxins entry and infection. One of the strategies that the host utilizes to avoid an inflammatory response against the microbiota is to use the intestinal barrier, including the mucous layer and immunoglobulin (Ig) A, an antibody isotype specialized in mucosal protection [2, 3] and produced locally by plasma cells present in the mucosal wall.

Previous research has shown that the process of weaning in piglets is associated with an increased inflammation response in the intestine [4, 5], and the potential negative effect of increased expression of inflammation markers on intestinal integrity [6, 7], morphology of intestinal structures such as villous length and crypt depth [4, 8] and disruption of the microbiota [9, 10], favoring a delay in intestinal maturation and a predisposal to diseases [11, 12]. Recent research by Pluske et al. [13] has shown the potential of different management measures around weaning such as implementing nutritional strategies to reduce intestinal pathogen load, increasing digestion and preventing production and activity of pro-inflammatory cytokines, all with the main goal to manipulate the immune system of pigs for improving performance, aiming to have an appropriate immune response for each specific circumstance, preventing to maximize the immune response [13]. Furthermore, nutritional strategies can modulate the complex interplay between the immune system and/or inflammatory responses and neuroendocrine mediators such as growth hormone and cortisol, thus having consequences on animal health and performances [14].

Promising examples of nutritional strategies are metabolites derived from edible mushrooms. Fungal fermented products and their derivatives are described to contain several compounds that may play a role in gastrointestinal health and pathogenic bacteria control [15]. Microbial enzymes produced by fungi during fermentation will degrade polysaccharides from feed material into indigestible and bioactive oligosaccharides [16]. *Agaricus subrufescens,* also known as *A. blazei murill* and/or the almond mushroom, is an edible mushroom, which grows naturally in Piedade, outside of São Paulo, Brazil. It contains high levels of biological response modulators, such as proteoglycans [17, 18] and β-glucans [19], which are a heterogeneous group of polysaccharides present in cereal grains, fungal cell walls, seaweed, and algae [20]. Products from *A. subrufescens* mushroom are reported to contain prophylactic and therapeutic properties including antimicrobial and immunomodulatory properties [15, 21].

The gastro-intestinal immune system is geared towards tolerance, in contrary to the systemic immune system, and responds to intestinal content (microbiota, metabolites, feed components, etc.), and this reaction can lead to tolerance (e.g. for commensal bacteria) or defense reactions [22]. One of the strategies that the host utilizes to avoid an inflammatory response against the microbiota is to use the intestinal barrier, including the mucous layer and immunoglobulin (Ig) A, an antibody isotype specialized in mucosal protection [2, 3]. Production of IgA is controlled by cytokine-producing T cells within the GALT and by cytokine released from the mucosa [2, 3, 23]. Previous studies report changes in the expression of inflammatory cytokines in the intestine of humans and animals during enteric infection and intestinal inflammatory diseases [24–26]. Both in vitro and in vivo investigations show that uncontrolled production of pro-inflammatory cytokines can influence gut integrity and epithelial functions, including permeability to macromolecules and transport of nutrients and ions [27]. Interactions of immune cell populations, present in the epithelium of the intestine, and other components of the intestinal mucosa are essential in the maintenance of symbiosis with commensals and the defense against pathogens.

From a nutritional perspective, controlling early intestinal inflammation is certainly a challenge in managing post-weaning gut disorders and preventing intestinal inflammation due to pathogenic enteric organisms can be an important pillar to maintain the health of piglets. The objective of this study was to evaluate the gastrointestinal health and immune modulation response induced by a blend of mannan-rich hydrolyzed copra meal and fermented rye with *A. subrufescens* in weaned piglets.

## Results

### Growth performance

Least squares means and standard errors for body weight (BW) and average daily gain (ADG) for control and Fysal® Solute (FS) treatment are given in Table 1. Average daily gain tended to be higher in FS treatment for day 0–15 (*P* = 0.078). There was no statistical evidence for other performance differences between control and treatment group.

**Table 1:** Effect of dietary treatment^1^ on performance of weaned piglets at different time points

	Day	Treatment	LSmeans	SEM	*P*-value
BW, kg	0	Control	5.59	0.225	0.705
		FS^1^	5.97	0.178	
	15	Control	8.84	0.761	0.111
		FS	10.4	0.603	
	30	Control	14.5	1.19	0.467
		FS	15.6	0.753	
	45	Control	17.9	2.12	0.318
		FS	20.7	1.84	
ADG, kg/d	0–15	Control	0.199^x^	0.043	0.079
		FS^1^	0.297^y^	0.034	
	0–30	Control	0.288	0.032	0.402
		FS	0.320	0.020	
	0–45	Control	0.305	0.035	0.605
		FS	0.330	0.031	
	30–45	Control	0.358	0.045	0.409
		FS	0.408	0.039	

SEM, standard error of the mean; BW, body weight; ADG, average daily gain

^x,y^Different superscripts within a column indicate a tendency difference (*P* < 0.10)

^1^Dietary treatment = additional Fysal Solute (FS) at 2 kg/ton

### Intestinal histomorphometry

**Fig. 1:**
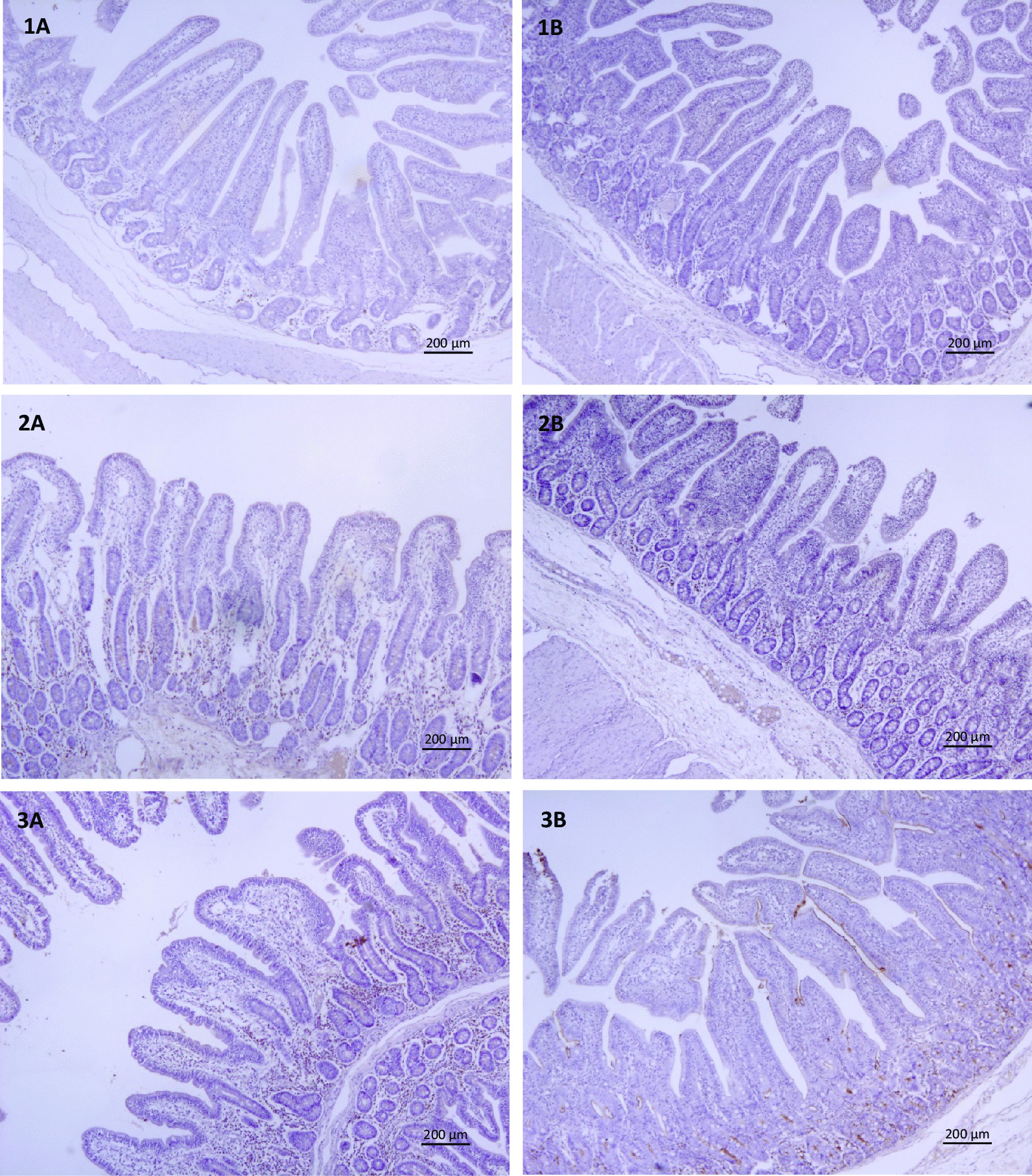
Immunohistochemical expression of IgA in the jejunum of animals from all experimental groups. The same sections were used, at different magnifications for histomorphometry and count of IgA producing cells. **1A**: Control day 15, **1B**: FS day 15, **2A**: Control day 30, **2B**: FS day 30, **3A** Control day 45, **3B** FS day 45

**Fig. 2:**
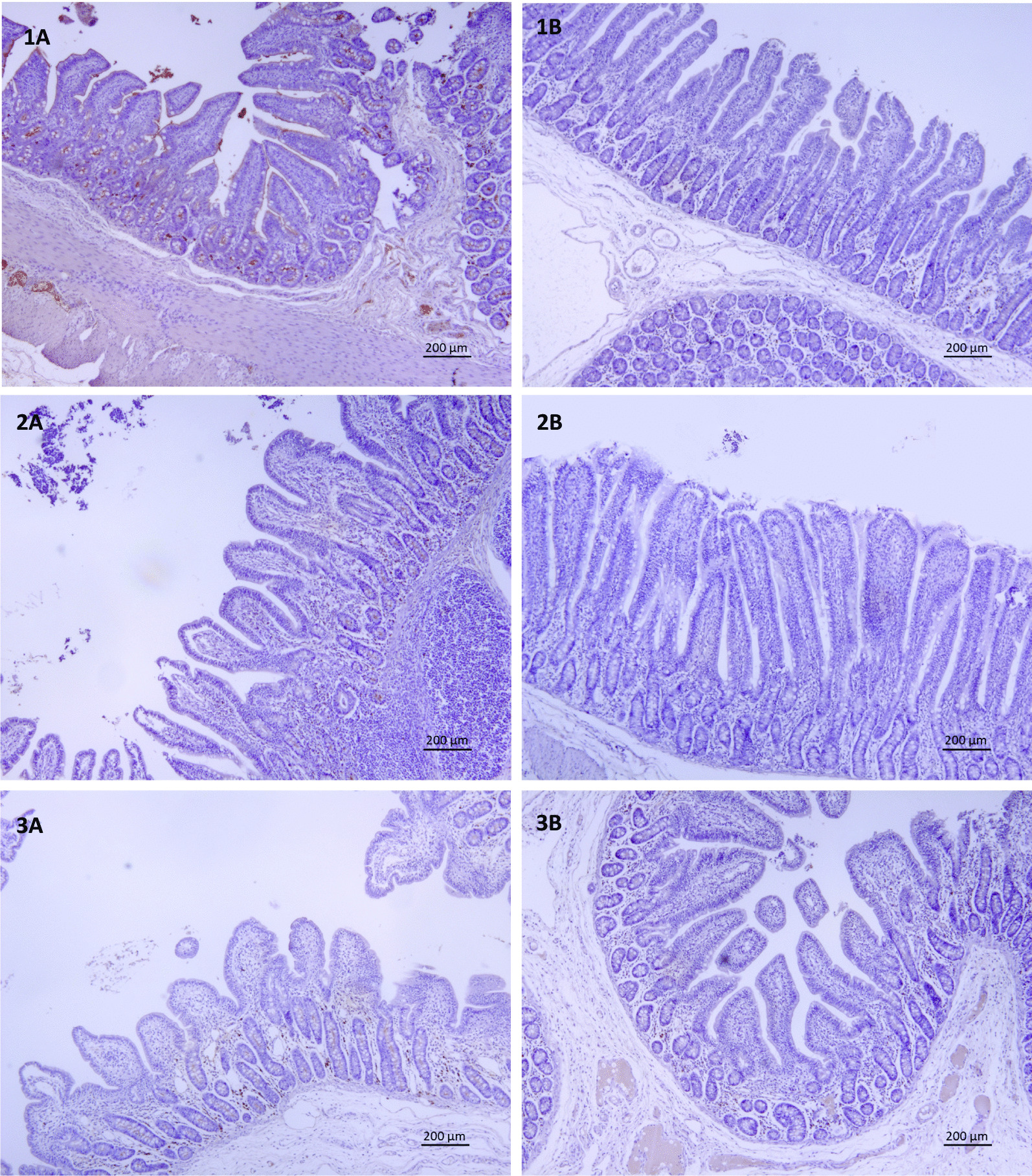
Immunohistochemical expression of IgA in the ileum of animals from all experimental groups. The same sections were used, at different magnifications for histomorphometry and count of IgA producing cells. **1A**: Control day 15, **1B**: FS day 15, **2A**: Control day 30, **2B**: FS day 30, **3A** Control day 45, **3B** FS day 45

Data on small intestine villi characteristics are presented in Table 2 and Figs. 1 and 2. The villous length in jejunal tissue was lower on day 15 for FS compared to control (*P* < 0.05). On day 15 in ileal tissue the villous length tended (*P* = 0.09) to be higher in FS compared to control. On day 30 an higher villous length in jejunal tissue (*P* < 0.05) and a tendency in ileal tissue (*P* = 0.09) were observed for FS compared to control. In ileal tissue the V/C ratio was higher in FS compared to control on day 30 (*P* < 0.05).

**Table 2:** Effect of dietary treatment^1^ on intestinal histomorphometry measurements in tissue of piglets at different time points

Day	Tissue	Parameter	Treatment	LSmeans	SEM	*P*-value
15	Jejunum	Villous length, μm	Control	492^a^	16.8	0.035
			FS^1^	438^b^	16.8	
		Crypt depth, μm	Control	292	13.7	0.264
			FS	317	16.0	
		V/C ratio^2^	Control	1.36	0.169	0.358
			FS	1.13	0.187	
	Ileum	Villous length, μm	Control	414^x^	15.7	0.093
			FS	452^y^	14.9	
		Crypt depth, μm	Control	258	17.0	0.116
			FS	297	17.0	
		V/C ratio	Control	1.50	0.104	0.657
			FS	1.57	0.104	
30	Jejunum	Villous length, μm	Control	447^a^	16.4	0.002
			FS	519^b^	10.6	
		Crypt depth, μm	Control	319	26.8	0.168
			FS	363	13.4	
		V/C ratio	Control	1.02	0.186	0.431
			FS	0.84	0.137	
	Ileum	Villous length, μm	Control	403^x^	26.6	0.093
			FS	459^y^	16.0	
		Crypt depth, μm	Control	354	29.8	0.356
			FS	320	18.0	
		V/C ratio	Control	1.18^a^	0.100	0.035
			FS	1.46^b^	0.068	
45	Jejunum	Villous length, μm	Control	516	25.4	0.969
			FS	515	22.8	
		Crypt depth, μm	Control	317	22.1	0.227
			FS	354	19.8	
		V/C ratio	Control	0.930	0.078	0.287
			FS	0.814	0.070	
	Ileum	Villous length, μm	Control	452	27.2	0.234
			FS	497	24.3	
		Crypt depth, μm	Control	324	16.3	0.428
			FS	305	17.3	
		V/C ratio	Control	1.49	0.100	0.717
			FS	1.55	0.100	

SEM, standard error of the mean

^a,b^Different superscripts within a column indicate a significant difference (*P* < 0.05)

^x,y^Different superscripts within a column indicate a tendency difference (*P* < 0.1)

^1^Dietary treatment = additional Fysal Solute (FS) at 2 kg/ton

^2^V/C ratio = Villous length (μm) divided by Crypt depth (μm)

### IgA producing cells in intestinal tissues

Several differences were observed between control and dietary treatment for various intestinal tissues and time points. Compared to control, the average count of IgA producing cells on day 15 was lower (*P* < 0.05) in piglets fed the FS diet in jejunum and ileum, being not different in colon. On day 30, compared to control, the average count of IgA producing cells was lower (*P* < 0.05) in piglets fed with FS in jejunum, ileum and colon. The average count of IgA producing cells in colon was lower (*P* < 0.05) in piglets fed with FS on day 45 compared to control (Table 3, Fig. 1), with no difference in jejunum and ileum on day 45.

**Table 3:** Effects of dietary treatment^1^ on Immunoglobulin A producing cells^2^ in tissue of piglets at different time points

Tissue	Day	Treatment	LSmeans	SEM	*P*-value
Jejunum	15	Control	13.6^a^	1.05	< 0.001
		FS	7.28^b^	1.00	
	30	Control	23.0^a^	1.60	0.009
		FS	17.3^b^	1.03	
	45	Control	26.0	3.00	0.544
		FS	23.5	2.72	
Ileum	15	Control	11.8^a^	0.896	0.001
		FS	6.62^b^	0.850	
	30	Control	20.2^a^	1.44	0.002
		FS	13.4^b^	1.02	
	45	Control	18.7	2.16	0.429
		FS	16.4	1.95	
Colon	15	Control	11.3	1.00	0.130
		FS	8.98	1.06	
	30	Control	19.7^a^	1.96	0.001
		FS	15.6^b^	0.91	
	45	Control	23.0^a^	1.31	0.012
		FS	17.9^b^	1.24	

SEM = standard error of the mean

^a,b^Different superscripts within a column indicate a significant difference (*P* < 0.05)

^1^Dietary treatment = additional Fysal Solute (FS) at 2 kg/ton

^2^Immunolabeled cells were counted in 10 non-overlapping consecutive high magnification fields of 25,000 μm

### Immunoglobulin concentration in serum

Comparing the effect of treatment on different immunoglobulin levels per sampling point, on day 45, a higher IgG quantity in blood was found in FS compared to control (*P* < 0.05). No differences were observed for IgE levels on any of the sampling time points. Dietary treatment FS had a higher IgA level in blood compared to control on day 15 (*P* < 0.05). On day 15, IgM concentration in blood tended to be higher in FS treatment (*P* = 0.07) compared to control. On day 45, higher IgM levels were observed in FS compared to control group (*P* < 0.05; Table 4).

**Table 4:** Effects of dietary treatment^1^ on immunoglobulin concentration in serum of piglets at different time points

Immunoglobulin	day	Treatment	LS means	SEM	*P*-value
IgG (mg/mL)	15	Control	5.89	0.829	
		FS	6.45	0.586	0.595
	30	Control	6.74	0.554	
		FS	7.65	0.429	0.243
	45	Control	7.67^a^	0.785	
		FS	9.94^b^	0.453	0.047
IgE (ng/mL)	15	Control	22.4	1.73	
		FS	25.0	1.41	0.277
	30	Control	22.0	4.36	
		FS	31.7	3.08	0.110
	45	Control	25.6	5.00	
		FS	21.0	2.67	0.443
IgA (ug/mL)	15	Control	72.4^a^	7.66	
		FS	101^b^	6.85	0.026
	30	Control	97.1	8.87	
		FS	107	6.27	0.371
	45	Control	108	41.2	
		FS	106	22.0	0.964
IgM (ug/mL)	15	Control	62.3^x^	21.3	
		FS	119^y^	15.1	0.066
	30	Control	114	17.5	
		FS	153	12.3	0.110
	45	Control	111^a^	11.5	
		FS	151^b^	6.15	0.019

SEM, standard error of the mean

^a,b^Different superscripts within a column indicate a significant difference (*P* < 0.05)

^x,y^Different superscripts within a column indicate a trend difference (*P* < 0.10)

^1^Dietary treatment = additional Fysal Solute (FS) at 2 kg/ton

### Cytokine gene expression in tissues

Several differences for cytokine expression were identified between dietary treatments. On day 15, a lower gene expression was observed for IFN-γ, IL-1α and IL-10 (*P* < 0.05) and a tendency (*P* < 0.10) for a lower expression of IL-1β, IL-6 and TNF-α in the jejunum. There were no differences on day 15 in ileum. In colon tissue, expression of IL-10 and IL-12β tended (*P* < 0.10) to be lower in FS compared to control. In PBMC on day 15, a significantly higher expression of IFN-α, IL-1α, IL-6, IL-10, IL-12β and TGF-β was shown in FS compared to control. Furthermore, tendencies for higher expression were shown for IL-1β, IL-8, IL-12α and TNF-α in FS treatment compared to control (*P* < 0.10; Table 5 and Appendix 1).

**Table 5:** Effects of dietary treatment^1^ on cytokine expression in tissues of piglets at different time points

Day	Tissue	IFN-α	IFN-γ	IL-1α	IL-1β	IL-6	IL-8	IL-10	IL-12α	IL-12 β	TNF-α	TGF-β
15	Jejunum		< *	< *	< ^	< ^		< *			< ^	
	Ileum											
	Colon							< ^		< ^		
	PBMC	> *		> *	> ^	> *	> ^	> *	> *	> *	> ^	> *
30	Jejunum	> ^										< ^
	Ileum							< ^				
	Colon							> *		> ^		
	PBMC											> ^
45	Jejunum		< *	< *				< *				
	Ileum						> *					
	Colon		< ^					< *		< ^		
	PBMC				< *							

(*) Significant (*P* < 0.05) and (^) tendency (*P* < 0.1) upregulation ( >) or downregulation ( <) of cytokine mRNA levels of FS compared to control

^1^Dietary treatment = additional Fysal Solute (FS) at 2 kg/ton

On day 30, tendencies were observed for a higher (*P* = 0.05) expression of IFN-α in the jejunum, and for a lower expression (*P* = 0.05) in TGF-β in FS treatment compared to control. In the ileum, a tendency indicated a lower expression of IL-10 and IL-12β (*P* < 0.10). In colon, a higher expression of IL-10 (*P* < 0.05) and a tendency for a higher expression in IL-12β in the FS treatment compared to control were observed (*P* = 0.089). In PBMC, a tendency for a higher TGF-β expression was observed in FS treatment compared to control (*P* = 0.07; Table 5 and Appendix 1).

On day 45, lower IFN-γ, IL-1α and IL-10 expression in the jejunum (*P* < 0.05), higher (*P* < 0.05) IL-8 expression in the ileum, lower (*P* < 0.05) IL-10 expression in colon were observed for FS treatment compared to control. FS tended (*P* < 0.1) to reduce IFN-γ and IL-12β expression in colon. Furthermore, in PBMC, IL-1β expression was (*P* < 0.05) reduced by FS compared to control (Table 5).

Comparing gene expression of cytokines in jejunum, ileum, colon and PBMC over the three time points, it was observed that FS treatment reduced local tissue gene expressions of several cytokines, mainly on day 15 and 45, and increases gene expression in PBMC mainly on day 15 (Table 5 and Appendix 1).

### Levels of cytokines and lymphocytes in blood

Differences between control and FS fed pigs in the concentration of IL-10 in blood are presented in Table 6. Pigs fed FS show a lower level on day 15 compared to control (*P* < 0.05), and a tendency for reduction on day 30 (*P* = 0.08; Table 6). No differences were observed in level of IFN-γ and the IFN-γ/IL-10 ratio between control and FS dietary treatments.

**Table 6:** Effects of dietary treatment^1^ on cytokine IFN-γ and IL-10 concentration and their ratio in blood of piglets at different time points

Day	Parameter	Treatment	LSmeans	SEM	*P*-value
15	IFN-γ, pg/mL	Control	7.55	2.91	0.189
		FS	12.9	2.38	
	IL-10, pg/mL	Control	11.9^a^	2.42	0.028
		FS	3.53^b^	1.98	
	Ratio IFN-γ/IL-10	Control	0.823	1.27	0.041
		FS	5.08	1.14	
30	IFN-γ, pg/mL	Control	4.48	0.816	0.377
		FS	3.54	0.577	
	IL-10, pg/mL	Control	51.3^x^	13.5	0.080
		FS	17.6^y^	9.52	
	Ratio IFN-γ/IL-10	Control	0.207	0.687	0.511
		FS	0.789	0.485	
45	IFN-γ, pg/mL	Control	3.26	1.34	0.648
		FS	3.99	0.720	
	IL-10, pg/mL	Control	6.46	2.28	0.934
		FS	6.24	1.22	
	Ratio IFN-γ/IL-10	Control	0.479	0.189	0.416
		FS	0.801	0.003	

SEM, standard error of the mean

^a,b^Different superscripts within a column indicate a significant difference (*P* < 0.05)

^x,y^Different superscripts within a row indicate a tendency difference (*P* < 0.1)

^1^Dietary treatment = additional Fysal Solute (FS) at 2 kg/ton

On day 15, level of NK cells was higher in FS compared to control (*P* < 0.05). On day 45, a lower level of B cells was observed in FS compared to control (*P* < 0.05). Th lymphocyte level tended to be lower on day 30 in pigs fed FS compared to control (*P* = 0.08). TCD3, a count for total T cells, level tended to be lower on day 15 in FS compared to control (*P* = 0.08). On day 45, CD4-CD8-lymphocytes were higher (*P* < 0.05), while CD4 + CD8 + levels were observed to be lower in FS compared to control (*P* = 0.07) (Table 7).

**Table 7:** Effects of dietary treatment^1^ on lymphocytes quantification^2^ in blood of piglets at different time points

Lymphocyte^3^	Day	Treatment	LSMeans	SEM	*P*-value
NK	15	Control	27.3^a^	1.48	0.035
		FS	32.2^b^	1.56	
	30	Control	23.3	4.737	0.278
		FS	29.3	3.09	
	45	Control	36.8	2.09	0.127
		FS	32.4	1.78	
B	15	Control	11.3	2.01	0.740
		FS	10.3	1.91	
	30	Control	12.3	1.45	0.142
		FS	9.55	1.03	
	45	Control	10.2^x^	0.917	0.069
		FS	7.85^y^	0.829	
T CD 4 (Th)	15	Control	25.3	1.86	0.555
		FS	23.7	1.86	
	30	Control	33.8^x^	2.81	0.081
		FS	27.4^y^	1.99	
	45	Control	27.1	1.25	0.849
		FS	27.4	1.13	
T CD8 (Tc)	15	Control	17.9	2.02	0.879
		FS	18.4	2.02	
	30	Control	20.9	2.04	0.944
		FS	20.6	1.70	
	45	Control	15.7	1.92	0.999
		FS	15.7	1.64	
TCD3	15	Control	62.3^x^	3.61	0.084
		FS	53.1^y^	3.42	
	30	Control	55.7	3.56	0.192
		FS	49.6	2.72	
	45	Control	50.5	2.74	0.991
		FS	50.5	2.49	

SEM, standard error of the mean

^a,b^Different superscripts within a column indicate a significant difference (*P* < 0.05)

^x,y^different superscripts within a column indicate a tendency difference (*P* < 0.1)

^1^Dietary treatment = additional Fysal® Solute (FS) at 2 kg/ton

^2^Measurement unit for all lymphocytes is amount of cells/μL

^3^NK = lymphocytes Natural killer (CD45 + CD3-CD16 + CD56 +), B = lymphocytes B (CD45 + CD21 +), T CD 4 Th = lymphocytes T helper (CD45 + CD3 + CD4a +), T CD 8 Tc = lymphocytes T cytotoxic (CD45 + CD3 + CD8a +), TCD3 = lymphocytes T (CD45 + CD3e +)

## Discussion

Weaning-associated intestinal inflammation occurs in piglets [4, 5]. Determining nutritional strategies to prevent intestinal inflammation due to pathogenic enteric organisms can be an important pillar to maintain health status of animals. The present study provides evidence in immune modulation response induced by a blend of mannan-rich hydrolyzed copra meal and fermented rye with *A. subrufescens* in piglets post-weaning.

The primary focus of the current study was on piglet immunity and intestinal health while due to design obvious reasons, the performance was secondary. Nonetheless, a tendency for improved ADG was observed in piglets fed FS during the first 15 days which suggests a potential benefit of FS effects on immunity and intestinal morphometry improving growth performance. Furthermore, throughout the study, no piglets developed diarrhea or other disease related clinical signs, indicating that this study was done in a healthy environment, authors suggest to repeat a similar study under higher disease pressure conditions.

There is little research assessing such fungi origin feed additive (or similar components) effect on pig performance. A report by the Danish National Committee for Pig Production described the effect of 0.1% *A. subrufescens* extract added to weaner feed, and found no differences in diarrhea or mortality, but an 11% increase in productivity in the supplemented group [28]. In a Chinese study, the use of *A. subrufescens* improved the growth performance of weaned piglets, with a reduced feed conversion rate [29]. Next to performance improvement, the researchers found that feeding soluble metabolites of *A. subrufescens* stimulated intestinal villous development, which was also found in more recent research, where the supplementation of *A. subrufescens* increased the villous height and the ratio of villous height/crypt depth of small intestine in mice [30]. These results imply that certain mycelial metabolites of *A. subrufescens* might be important in enhancing villous development, which might consequently enhance the absorption function of intestine. The use of FS in current study lowered villous height on day 15 in jejunum, while villous height in ileum on that same time point was higher, and higher villous height was seen on day 30 both in jejunum as in ileum. Peculiar about these results was that the lower villous height on day 15 in jejunum coincides with a tendency for improvement in growth performance. In fact, the villous height, crypt depth and their ratio in the small intestine are indexes to determine the capacity of intestinal absorption [31] and usually associated with an improvement in growth performance [32]. Nonetheless, there is also evidence that in germ-free animals a slower turnover rate of intestinal epithelial cells resulted in longer villi [33]. A slower turnover is seen in neonatal pigs, with an epithelial cell turnover of 7 to 10 days compared to a 2- to 3-day turnover when pigs are 3 weeks of age [34]. Extensively studied are the effects of weaning age on intestinal morphology in the piglets, where early weaning causes villous shortening [35–37]. Therefore, further studies may be required to clarify the mechanism of this improvement by FS on intestinal integrity and growth performance, in different commercial practices such as varying age of weaning genetics, which is known to influence the immune system in pigs [38–40].

The quantity of IgA producing cells in jejunum and ileum on days 15 and 30 and in colon on days 30 and 45 was reduced in piglets fed FS. A potential explanation for this reduction in IgA producing cells could be a lower stimulation by antigens in the pigs intestine. Antigens stimulate mucosal plasma cells to secrete IgA, causing a response in mesenteric lymph nodes, which increases the number of IgA expressing cells [3]. Reduced intestinal IgA producing cells in piglets fed FS coincided with elevated IgA and IgM levels in serum on day 15. It is generally accepted that, after antigenic stimulation in the Peyer's patches, IgA + lymphoblasts migrate through the lymph and blood circulation to the lamina propria of the intestine. In the current study it could be hypothesized that the lack of antigenic stimulation in the intestine of piglets fed FS not only reduces the response for increasing number of IgA producing cells in the intestinal tissue but also prevents migration of IgA cells towards the lamina propia in the intestine, potentially explaining the higher levels of IgA in the blood of piglets fed FS. Further research is needed to prove this hypothesis, where the focus should be combining this work with extensive microbiota analysis. Previous research performed in piglets has shown a significant decrease in piglet’s plasma IgG concentrations immediately after weaning, which coincides with the depletion of maternal immunity [5, 7, 41]. In current study, piglets receiving FS had an increase in serum IgG quantity at day 45. Previous in vivo results have shown the extract of the mushroom exhibited a significantly increased serum IgG level, T-cell population and phagocytic activity [42] in mice.

Analyses on lymphocyte concentration in blood demonstrated an increase in NK cells when feeding FS component. Interestingly, previous studies with the same mushroom fraction also enhanced the activity of NK cells in spleen [43, 44]. Yuminamochi et al. [45] demonstrated that powdered dried fruiting bodies and hemicellulase-digested component of *A. subrufescens* augmented NK cells activation through IL-12 mediated IFN-γ production. β-glucans and proteoglucans are known to be potent stimulators of macrophages [46–48], polymorph nuclear neutrophils (PMN) [49] and Natural Killer (NK) cells [50]. In current study, besides an increase in amount of NK cells, lower amounts of T and B cells were observed in the treatment group at different timepoints. This can be of interest due to the previously reported downregulation of T helper pro-inflammatory pathways by β-1,4 mannobiose in a colitis pig model, induced by dextran sodium sulfate at 1.25 g/kg BW/day, which maintained intestinal permeability and histological morphology [51]. The immunomodulatory properties of different β-glucans have been demonstrated in vitro [21, 52]. Recent studies in pigs have shown modulation of mucosal immunity by binding of polysaccharides to specific receptors of immune cells. This could provide beneficial effects on animal health and resistance to disease, since blocking fimbriae of pathogenic bacteria prevents their adhesion to the mucous epithelium which may prevent or eliminate infection [53]. A challenge study induced by F18 *Escherichia coli* (10^10^ cfu/3 ml for 3 days) demonstrated a reduction in diarrhea, potentially caused by a reduced gut permeability, together with a reduction in mRNA expression of IL-1β, IL-6, and TNF-α in ileal mucosa of piglets receiving β-glucans in their diets [54].

Current study shows, that next to a reduction in IgA producing cells in the intestine, lower intestinal cytokine expression was seen in piglets fed FS, mainly in expression of IFN-γ, IL-1α and IL-1β, IL-6, IL-10 and TNF-α. Previous research has shown that challenging conditions can increase the gene expression of pro-inflammatory cytokines, such as the process of weaning or bacterial or infectious challenges. Studies provide evidence of cytokine regulation by weaning process with increased production of pro-inflammatory cytokines post-weaning [4, 5, 55]. Production of intestinal mucosal IgA is controlled by cytokine-producing T cells within the GALT and by cytokines released from the mucosa. Within the GALT, the Th_1_ cytokines, IFN-γ and TNF-β, downregulate IgA production, whereas the Th_2_ cytokines, IL-4, IL-5, IL-6, and IL-10, upregulate IgA production [56, 57]. Several cytokines, such as TGF-β, IL-1α, and IL-6 are constitutively expressed by the intestinal epithelium and may play a role in the normal influx of immune cells into the mucosa, in the growth of epithelial cells and in homeostasis [58]. Resulting from a study performed in humans, other cytokines, such as IL-8, IL-1β and TNF-α are also expressed by normal epithelial cells but are upregulated in response to microbial infection [59]. The reduction in IgA secretory cells, combined with lower expression of cytokines in the intestinal mucosa could implicate a lower stimulated immune system, implicating a potential immunomodulating effect of FS.

This potential mode of action of fungal fermented products and their derivatives has been previously reported, where they are described to contain several compounds that may play a role in gastrointestinal health and pathogenic bacteria control [15]. Promising examples are metabolites derived from edible mushrooms. Products from the *A. subrufescens* are reported to contain prophylactic and therapeutic properties, including antimicrobial and immunomodulatory properties [15, 21]. *A. subrufescens,* also known as *A. blazei murill*, is an edible mushroom, which grows naturally in Piedade, outside of São Paulo, Brazil. It contains high levels of biological response modulators, such as proteoglycans [17, 18] and β-glucans [19], which are a heterogeneous group of polysaccharides present in cereal grains, fungal cell walls, seaweed, and algae [20]. Microbial enzymes produced by fungi during fermentation will degrade polysaccharides from feed material into indigestible and bioactive oligosaccharides [16].

Many successful enteric organisms have developed strategies to resist displacement from the epithelium via the development of anchoring adhesive fimbriae (pili) [60]. Approaches to masking these attachment sites from pathogenic bacteria include the feeding of competing carbohydrates (oligosaccharides) that inhibit attachment of certain bacteria to the epithelium [61]. Adhesins from some pathogenic *Enterobacteriaceae* are known to show binding affinity to distinct indigestible oligosaccharides. Wang et al. [62] demonstrated such affinity for suitable oligosaccharides, i.e., d-mannose showed between 20 and 60% inhibition of bacterial adhesion (*E. coli*, *Vibrio cholerae*, *Campylobacter jejuni*, and *Salmonella* t*yphimurium*) to host glycans from HT-29 cells (54). For example, addition of 2–5% D-mannose to broiler diets reduced the excretion and colonization of *S. enterica* var. *typhimurium* [63], while the use of a mannan oligosaccharide has been reported to reduce the concentration of caecal coliforms and *S. enterica* var. *typhimurium* and *S. dublin* in chicks [60, 64]. A recent study demonstrated the effect of in vitro binding of FS, a blend of mannan-rich hydrolyzed copra meal, containing levels of mannobiose, and a fungal fermented rye with *A. subrufescens*, to *S. enterica* serovars *typhimurium* and *enteritidis*. In vivo results demonstrated that feeding those components to nursery pigs reduced the peak and average *S. typhimurium* shedding compared with control [65]. Based on the recent data with FS and its components, in current study the potential beneficial effect of FS against pathogenic bacteria by its binding capacity could have lowered the number of pathogenic bacteria in the lumen, or shifting microbiota reducing an immunological response initiated by the dendritic cells in the intestine. This can in turn result in a lower amount of IgA producing cells and a lower gene expression of cytokines such as IFN-γ, IL-1, IL-6, IL-10 and, IL-12 and TNF-α in the intestine. However, current study lacks data on the response of the intestinal microbiota to feeding component FS, therefore the exact mode of action of the lower intestinal inflammation response remains to be clarified.

Previous research done using the FS component in combination with organic acids indicated the potential in modifying the intestinal microbiota, with a higher abundance of *Lactobacillus spp* and a lower abundance of *Clostridium spp.* after a *S. typhimurium* challenge in post-weaning piglets [66]. Furthermore, previous work in grower pigs showed that fed a dietary supplementation of *A. bisporus* (white button) mushrooms positively affected the composition of the fecal and proximal colon microbiota by promoting the abundance of *Ruminococcaceae* and *Lachnospiracea* families [67]. These families are known for degradation of complex plant material (cellulose and hemicellulose) in the mammalian gut and are considered as beneficial given their production of butyrate [68]. The *A. bisporus* mushrooms fed to pigs did not affect growth rate, intestinal permeability or systemic and localized activation of mononuclear cells [67]. However, an anti-inflammatory effect was observed in LPS-stimulated alveolar macrophages with a significant reduction in IL-1β gene expression and cytokine production reflected in a lower activation of IL-1-signaling in pigs fed *A. bisporus* mushrooms.

Current study showed that reduced intestinal cytokine gene expression coincided with elevated cytokine gene expression in PBMC. This is in agreement with previous research where the use of *A. subrufescens* is shown to stimulate cytokine production in PBMC, such as interleukin-12 (IL-12) [43], or interferon-γ (IFN-γ) [45]. Furthermore, the β-1,6-d-glucan fraction extracted from *A. subrufescens* induces IFN-γ production and can partially reverse the production of IL-10 [69], the latter is in agreement with results of current study. *A. subrufescens* fraction had been shown to induce macrophages to secrete (TNF-α), (IL)-8 and nitric oxide (NO) in an in vitro test [70]. Research in human medicine showed *A. subrufescens* extract to promote anti-inflammatory effects without side effects. Like in current study, pro-inflammatory cytokines (IL-1β and IL-6) and chemokine (IL-8) were downregulated, and this was demonstrated ex vivo with heparinized blood of exposed Colitis Ulcerosa, Crohn’s Disease and normal patients [71]. These relatively common human diseases are characterized and a consequence of deficiency or enhanced activation of cytokine pathways, which promotes the breakdown of intestinal homeostasis [72, 73]. In pigs, more research is needed in this area. However, the reported post-weaning intestinal upregulation of pro-inflammatory cytokine production [4, 5] demonstrates the potential of implementing nutritional strategies as shown in current study, with the aim to manipulate the immune system of pigs and reduce the immune response.

## Conclusion

In post-weaning piglets, feeding component FS, a blend of mannan-rich hydrolyzed copra meal and fermented rye with *A. subrufescens,* stimulates an immune modulation effect most evident at 15 days post-weaning. The effect includes a reduction of local intestinal inflammatory response, with emphasis in jejunum, accompanied with an increase in systemic (PBMCs) cytokine gene expression and a higher villous height in jejunum and ileum on day 30, while it was observed to be lower in jejunum on day 15.

## Methods

### Animals, housing, and experimental design

Current study was performed at the Veterinary Teaching Farm of the University of Murcia (Spain), using 72 piglets (Large White) weaned at 22 ± 3 days (d) of age with an average initial body weight (BW) of 5.53 ± 1.19 kg and about 1:1 male:female ratio. Piglets were obtained from 12 different litters from sows with average parity of 3.43. Without receiving any feed before weaning, after weaning all pigs were allotted visually trying to avoid great difference in weight among the animals in the same pen. Pens were randomly allotted to two treatments with 4 pens per treatment and 9 animals per pen (pen size: 0.61 × 1.22 m). Pigs were housed in an environmentally controlled unit (25–27 °C) with natural light throughout the experiment. The pens were full-slated (plastic slat) and contained one nipple drinker ad libitum and one feeder with 4 spaces. On day 4 post-weaning piglets were routinely vaccinated against PCV2 (Porcilis PCV-2, MSD) and no systematic medications were included in feed or water.

### Diets and additives

A two-phase experimental diet was used, produced at a local feedmill (Pigalomar; Spain), without additional additives or medication but the dietary treatments reported (Table 8). All diets were pelleted (4 mm) and were formulated to meet the current estimates for nutrient growth requirements for nursery pigs [74]. Diets did not contain spray-dried plasma, antibiotics, and pharmaceutical levels of zinc oxide. The dietary treatments consisted of a control diet and a treatment diet, being the control diet + 0.2% Fysal® Solute (FS), a feed additive consisting of a blend of mannan-rich hydrolyzed copra meal and fermented rye with *A. subrufescens*. The feed additive used in the present experiment was provided by Trouw Nutrition, The Netherlands.

**Table 8:** Composition of the experimental diets

Item	Phase 1 Day 0–14	Phase 2 Day 15–45
*Ingredients, %*		
Barley	29.98	25.00
Wheat	24.00	26.89
Corn	17.17	19.50
Soybean Meal 47 crude protein	6.00	16.83
Ca carbonate	0.45	0.61
Monocalcium phosphate	0.75	0.78
Soybean oil	3.50	3.67
Intestinal swine mucosal hydrolyzate^1^	2.50	0.00
Milkpowder	5.00	0.00
Fysal MP^2^	0.30	0.30
Salt	0.30	0.44
L-Valine (96.5%)	0.050	0.025
DL-Methionine (99%)	0.175	0.175
L-Lysine HCl (98%)	0.542	0.525
L-Threonine (98%)	0.258	0.250
L-Tryptophan (98%)	0.033	0.008
Protein concentrate^3^	6.00	2.00
Trouwmix 30 premix^4^	3.00	3.00

^1^Protein source as hydrolysed peptides from porcine intestinal mucosa

^2^Blend of free and buffered organic acids (Formic, acetic, propionic acid)

^3^Gluten meal, extruded soybean meal, potato protein

^4^Vitamin and mineral premix provided the following per kilogram of diet: vitamin A, 15,000 IU; vitamin D, 2000 IU; vitamin E, 100 IU; 30 μg of vitamin B12; vitamin K, 2 mg; D-pantothenicacid 15 mg as calcium pantothenate; 30 mg of nicotinicacid; choline, 150 mg as betaine hydrchloride; Mn, 50 mg as manganese oxide; Zn, 105 mg as zinc oxide; Fe, 100 mg as iron sulphate; Cu, 120 mg as copper sulphate; I, 1.5 mg as potassium iodide; Se, 0.42 mg as sodium selenite; 6-phytase 1500 Phytase Unit (FTU)

### Growth performance and sample collection

Individual body weight of piglets was recorded at day 0 (weaning), day 15, day 30 and day 45, and average daily gain was calculated. At weaning (weaning + 18 h), 10 control animals were sacrificed to obtain blood from the vena jugular and intestinal tissue samples as a basal treatment. Animals were euthanized by lethal intravenous injection with an overdose of tiobarbital IV (50 mg/kg BW, Tiobarbital Braun Medical S.A., Barcelona, Spain). Subsequently, sacrification was done randomly at day 15 (control n = 11, FS n = 10), day 30 (control n = 6, FS n = 12) and day 45 (control n = 9, FS n = 13) to obtain blood and intestinal tissue samples. Tissue samples were obtained from the jejunum (middle section), ileum (5 cm adjacent to ileocecal valve), and colon (apex section of spiral) by three centimeter long gut sections, opened along the mesenterial insertion and 2 cm cut from their middle section. After been gently washed in water, each sample was divided in two subsamples, one (20 mg) was preserved in RNAlater (Life Technologies, USA) and one was fixed in 10% buffered formaldehyde and embedded in paraffin-wax for histomorphometrical and immunohistochemical studies. Blood samples were collected into ethylenediaminetetraacetic (EDTA) tubes (Vacutainer, Becton Dickinson, UK). Peripheral blood mononuclear cells (PBMCs) were isolated by Histopaque gradient and preserved in RNAlater (Life Technologies, USA) at − 80 °C, after 24 h of refrigeration at 8 °C, for subsequent gene expression analysis. Serum and plasma were isolated from whole blood by centrifugation (251 rcf, 10 min at room temperature) and preserved at -80 °C up to analysis.

### Sample analysis

#### Blood immunoglobulin analysis

Total serum immunoglobulins IgA, IgE, IgG and IgM were quantified in an amount of 50 μL serum sample by Enzyme Linked Immuno Sorbent Assay (ELISA) Porcine IgA (Immunoglobulin A) ELISA Kit E-EL-P1273; Porcine IgE (Immunoglobulin E) ELISA Kit E-EL-P0286; Porcine IgG (Immunoglobulin G) ELISA Kit E-EL-P0004; and Porcine IgM (Immunoglobulin M) ELISA Kit E-EL-P2269 (Elabscience, USA) per manufacturer instructions. For establishment of the immunotype Th1/Th2, the cytokines IL-10 and IFN-γ have been quantified in 50 μL of serum sample by means of ELISA (Invitrogen, USA) as per manufacturer instructions.

#### B cells, T cells and NK cells

Flow cytometry was used to analyse subpopulations of different types of lymphocytes: B cells (CD45 + CD21 +), NK (CD45 + CD3e-CD16 + CD56 +), T lymphocytes (CD45 + CD3e +), helper T lymphocytes (Th) (CD45 + CD3e + CD4a + CD8a-), cytotoxic T lymphocytes (Tc) (CD45 + CD3e + CD4a-CD8a +). A sample of peripheral blood per tube was mixed with antibodies, using mouse anti-human CD56:RPE (clone MEM-188) (BioLegend, CA, USA), mouse anti-pig CD16:RPE (clone G7) and mouse anti-pig CD45:FITC (clone K252.1E4) (Bio-RAD, CA, USA), mouse anti-pig CD4a:PerCP-Cy5.5 (clone 74–12-4 or PT4), mouse anti-pig CD8a:PE (clone 76–2-11 or PT8), mouse anti-pig CD3e:PE-Cy7 (clone BB23-8E6-8C8) and mouse anti-human CD21:PE-Cy5 (clone B-ly4) (BD Pharmingen, Becton, Dickinson and Company, NJ, USA). Due to lack of porcine antibodies, human CD21 and CD56 were chosen based on cross-reactivity and guaranty for use in pigs. CD45 was used to differentiate the population of lymphocytes from the rest of the leukocytes (monocytes and granulocytes). Two tubes were prepared per sample, with tube 1 containing 50 µL of peripheral blood with 5 μL CD45 and 2.5 μL CD3, CD8 and CD4. Tube 2 contained 50 µL of peripheral blood with 5 μL CD45 and CD16, 2.5 μL CD3 and CD56 and 10 μL CD21. After mixing and 15 min of incubation, the sample was lysed with a 0.5 ml lysin solution (FACS LYSING, Becton, Dickinson and Company, NJ, USA). After mixing and 15 min of incubation, the sample was analysed in the FC500 cytometer (Beckman Coulter, IN, USA) by using a single 488 nm (blue) laser and obtaining analysis matrices for each analyte.

#### Immunohistochemistry for levels of IgA producing cells

In jejunum, ileum and colon tissue IgA secretory cells were detected using the avidin-biotine-peroxidase complex technique. From the samples embedded in paraffin-wax 5 μm thick slides were obtained, dewaxed and dehydrated with graded ethanol and the endogenous peroxidase activity was quenched in 3% H_2_O_2_ in methanol for 30 min. Samples were treated with 10% pronase in TBS (Sigma-Aldrich, USA) for antigen retrieval (12 min). After pretreatment, samples were rinsed three times in TBS for 5 min each and incubated for 30 min with 100 μL of blocking solution per slide at room temperature in a humid chamber, before incubation for 1 h at 37 °C with the primary antibody goat anti-pig IgA (A100-102, Bethyl, USA) diluted 1:3000 in TBS. The secondary antibody biotin conjugate rabbit anti-goat Ab (Dako, USA) diluted 1:300 in TBS was incubated for 30 min at room temperature. The Vectastain® Elite ABC kit (Vector, USA) was applied for 1 h at room temperature. Positive labeling was detected using 3,3′-diaminobenzidine tetrahydrochloride (Dako, USA). Sections were counterstained with Mayer's haematoxylin, dehydrated and mounted.By using a Zeiss Axioskop 40 microscope (Carl Zeiss, Oberkochen, Germany) with a Spot Insight camera and the Spot Advanced software (Spot Imaging Solution, Michigan, USA) the number of IgA secretory cells in the intestinal lamina propria was counted. Immunolabeled cells were counted in 10 non-overlapping consecutive high magnification fields of 25.000 μm^2^.

#### Cytokine gene expression

Gene expression for cytokines IFN-α, IFN-γ, IL-1α, IL-1β, IL-6, IL-8, IL-10, IL-12p35 (IL-12α), IL-12p40 (IL-12β), TNF-α, and TGF-β was analysed using relative quantification, with primers previously described in literature (see Table 9). Total RNA was isolated from tissue and PBMCs samples by means of Micro RNeasy kit (Qiagen, USA) and DNAc was synthetized using the Geneamp RNA PCR Core Kit (Life Technology, USA). The PCRs were performed using a 7300 ABI thermocycler (Life Technologies, USA) and the GoTaq® qPCR Master Mix (Promega, USA) with SYBR-Green chemistry. The specificity of the reaction was assessed by analyzing the melting curve. The samples were normalized using the average Ct for glyceraldehyde-3-phosphatedehydrogenase (GAPDH), cyclophilin and β-Actin. The expression for each sample was calculated [84], correcting to the PCRs efficiency, calculated by serial decimal dilutions and using the slope offered by the thermacycler software, and used as control group the animals sampled at day 0. The efficiency of q-PCRs was between 91 and 105%. Data were expressed as fold change, normalized to the lowest value (value = 1).

**Table 9:** Primers of the cytokines IFN-α, IFN-γ, IL-1α, IL-1β, IL-6, IL-8, IL-10, IL-12p35 (IL-12α), IL-12p40 (IL-12β), TNF-α and TGF-β and primers of glyceraldehyde-3-phosphatedehydrogenase (GAPDH), cyclophilin and β-actin

	Primer forward (5′ → 3′)	Primer reverse (5′ → 3′)	References
IFN-α	5′-CCCCTGTGCCTGGGAGAT-3′	5′-AGGTTTCTGGAGGAAGAGAAGGA-3′	[75]
IFN-γ	5′-TGGTAGCTCTGGGAAACTGAATG-3′	5′-GGCTTTGCGCTGGATCTG-3′	[76]
IL-1α	5′-GTGCTCAAAACGAAGACGAACC-3′	5′-CATATTGCCATGCTTTTCCCAGAA-3′	[77]
IL-1β	5′-AACGTGCAGTCTATGGAGT-3′	5′-GAACACCACTTCTCTCTTCA-3′	[78]
IL-6	5′-CTGGCAGAAAACAACCTGAACC-3′	5′-TGATTCTCATCAAGCAGGTCTCC-3′	[78]
IL-8	5′-GCTCTCTGTGAGGCTGCAGTTC-3′	5′-AAGGTGTGGAATGCGTATTTATGC-3′	[79]
IL-10	5′-TGAGAACAGCTGCATCCACTTC-3′	5′-TCTGGTCCTTCGTTTGAAAGAAA-3′	[77]
IL-12p35	5′-AGTTCCAGGCCATGAATGCA-3′	5′-TGGCACAGTCTCACTGTTGA-3′	[75]
IL-12p40	5′-TTTCAGACCCGACGAACTCT-3′	5′-CATTGGGGTACCAGTCCAAC-3′	[80]
TNF-α	5′-ACTCGGAACCTCATGGACAG-3′	5′-AGGGGTGAGTCAGTGTGACC-3′	[81]
TGF-β	5′-CACGTGGAGCTATACCAGAA-3′	5′-TCCGGTGACATCAAAGGACA-3′	[76]
β-actin	5′-CTACGTCGCCCTGGACTTC-3′	5′-GATGCCGCAGGATTCCAT-3′	[82]
Cyclophilin	5′-TGCTTTCACAGAATAATTCCAGGATTTA-3′	5′-GACTTGCCACCAGTGCCATTA-3′	[83]
GAPDH	5′-ACATGGCCTCCAAGGAGTAAGA-3′	5′-GATCGAGTTGGGGCTGTGACT-3′	[83]

#### Intestinal histomorphometry

Five photomicrographs were taken with a Zeiss Axiocam 503 color (Carl Zeiss, Oberkochen, Germany) coupled to a Zeiss Axioskop 40 microscope (Carl Zeiss, Oberkochen, Germany) with 10 × magnification, from each section of jejunum and ileum. Both the height of the villous (tip to villous-crypt junction) and depth of the crypt (from villous-crypt junction to the base of villous) were analysed with the ZEISS Efficient Navigation software (Carl Zeiss, Oberkochen, Germany), according to manufacturer instruction. Randomly selected well-oriented intact villi and crypts (n = 10) were measured per piglet, per timepoint for jejunal and ileal tissue. The mean villous height and crypt depth of each intestinal tissue was calculated and by dividing villous height by crypt depth the villous height/crypt depth ratio was calculated. All morphometric measurements were performed by the same blinded to treatments researcher.

### Statistical analysis

Data were analyzed using the MIXED procedure of SAS (version 9.4, SAS Institute; Cary, USA). The cytokine gene expression data was transformed as Log2 of the value for normalization. The model included the fixed effects of treatment. Animal was the experimental unit. Statistical significance and tendency were considered at *P* ≤ 0.05 and 0.05 ≤ *P* ≤ 0.10, respectively.

## Supplementary Information

**Additional file 1**. **Appendix. Table 1.** Effects of treatment^1^ on cytokine expression in jejunum, ileum and colon tissue of piglets at day 15. **Table 2.** Effects of treatment^1^ on cytokine expression in jejunum, ileum and colon tissue and PBMC of piglets at day 30. **Table 3.** Effects of treatment on cytokine expression in jejunum, ileum and colon tissue of piglets at day 45.

## Data Availability

The datasets supporting the conclusions of this article are included within the article (and its Additional file 1 (Appendix).
