# Supplementary material for: Fermented rye with Agaricus subrufescens and mannan-rich hydrolysate based feed additive to modulate post-weaning piglet immune response
Source: Porcine Health Manag. 2021 Dec 9;7:60. doi: 10.1186/s40813-021-00241-y (PMC8656036; doi:10.1186/s40813-021-00241-y)
Supplement: Supplementary file 1 — Additional file 1. Appendix. Table 1. Effects of treatment1 on cytokine expression in jejunum, ileum and colon tissue of piglets at day 15. Table 2. Effects of treatment1 on cytokine expression in jejunum, ileum and colon tissue and PBMC of piglets at day 30. Table 3. Effects of treatment on cytokine expression in jejunum, ileum and colon tissue of piglets at day 45. [file 40813_2021_241_MOESM1_ESM.docx]

**Appendix**

**Table 1** - Effects of treatment^1^ on cytokine expression in jejunum, ileum and colon tissue of piglets at day 15

|  |  | Jejunum | | | Ileum | | | Colon | | | PBMC | | |
| --- | --- | --- | --- | --- | --- | --- | --- | --- | --- | --- | --- | --- | --- |
|  | Treatment | LS Means | SEM | P-value | LS Means | SEM | P-value | LS Means | SEM | P-value | LS Means | SEM | P-value |
| IFN-α | Control | 2.32 | 0.346 | 0.229 | 3.34 | 0.681 | 0.252 | 1.75 | 0.183 | 0.978 | 10.4^b^ | 1.81 | 0.002 |
|  | FS | 1.72 | 0.328 |  | 2.22 | 0.646 |  | 1.74 | 0.183 |  | 20.7^a^ | 1.92 |  |
| IFG-γ | Control | 5.03^a^ | 0.427 | 0.021 | 8.12 | 0.367 | 0.845 | 3.10 | 0.115 | 0.498 | 14.5 | 3.21 | 0.417 |
|  | FS | 3.50^b^ | 0.427 |  | 8.02 | 0.367 |  | 2.99 | 0.109 |  | 18.3 | 3.21 |  |
| IL-1α | Control | 5.43^b^ | 0.216 | 0.039 | 7.53 | 0.251 | 0.465 | 8.72 | 0.866 | 0.216 | 4.0 | 1.98 | 0.004 |
|  | FS | 4.75^a^ | 0.216 |  | 7.81 | 0.265 |  | 8.42 | 0.866 |  | 12.9 | 1.75 |  |
| IL-1β | Control | 5.65^y^ | 0.444 | 0.105 | 5.74 | 0.673 | 0.167 | 4.26 | 0.381 | 0.361 | 14.1^y^ | 2.00 | 0.063 |
|  | FS | 4.60^x^ | 0.421 |  | 4.40 | 0.638 |  | 3.76 | 0.381 |  | 19.8^x^ | 2.00 |  |
| IL-6 | Control | 2.36^y^ | 0.336 | 0.056 | 3.63 | 0.431 | 0.766 | 3.57 | 0.297 | 0.130 | 14.7^a^ | 1.21 | 0.027 |
|  | FS | 1.36^x^ | 0.354 |  | 3.82 | 0.454 |  | 2.89 | 0.313 |  | 18.9^b^ | 1.21 |  |
| IL-8 | Control | 2.16 | 0.216 | 0.120 | 2.85 | 0.286 | 0.255 | 4.67 | 0.412 | 0.151 | 18.6 | 2.59 | 0.094 |
|  | FS | 1.66 | 0.216 |  | 2.38 | 0.286 |  | 3.80 | 0.412 |  | 25.0 | 2.44 |  |
| IL-10 | Control | 3.98^a^ | 0.230 | 0.022 | 3.26 | 0.030 | 0.205 | 3.73^y^ | 0.304 | 0.065 | 10.4^a^ | 1.03 | <.0001 |
|  | FS | 3.11^b^ | 0.258 |  | 3.20 | 0.032 |  | 2.92^x^ | 0.304 |  | 23.2^b^ | 1.17 |  |
| IL-12α | Control | 7.29 | 0.758 | 0.749 | 3.71 | 0.806 | 0.345 | 4.33 | 0.446 | 0.363 | 13.2^a^ | 2.12 | 0.010 |
|  | FS | 6.94 | 0.758 |  | 2.57 | 0.850 |  | 4.90 | 0.423 |  | 22.0^b^ | 2.12 |  |
| IL-12β | Control | 5.13 | 0.217 | 0.315 | 2.58 | 0.319 | 0.312 | 3.23^x^ | 0.286 | 0.077 | 16.5^b^ | 1.63 | 0.019 |
|  | FS | 4.81 | 0.229 |  | 3.06 | 0.336 |  | 2.44^y^ | 0.302 |  | 22.4^a^ | 1.54 |  |
| TNF-α | Control | 11.5^x^ | 1.19 | 0.078 | 12.5 | 1.31 | 0.186 | 20.6 | 0.727 | 0.489 | 15.8^y^ | 1.69 | 0.056 |
|  | FS | 8.42^y^ | 1.13 |  | 9.88 | 1.38 |  | 19.8 | 0.727 |  | 20.6^x^ | 1.59 |  |
| TGF-β | Control | 13.0 | 0.248 | 0.455 | 10.9 | 0.330 | 0.662 | 11.5 | 0.205 | 0.482 | 9.5^a^ | 1.74 | 0.001 |
|  | FS | 12.73 | 0.261 |  | 11.1 | 0.314 |  | 11.3 | 0.205 |  | 19.6^b^ | 1.85 |  |

Note: SEM = standard error of the mean. ^a–b^ Different superscripts within a row indicate a significant difference (*P*<0.05), ^x-y^ different superscripts within a row indicate a tendency difference (*P*<0.1), ^1^Dietary treatment = additional Fysal^®^ Solute (FS) at 2 kg/ton

**Table 2** - Effects of treatment^1^ on cytokine expression in jejunum, ileum and colon tissue and PBMC of piglets at day 30

|  |  | Jejunum | | | Ileum | | | Colon | | | PBMC | | |
| --- | --- | --- | --- | --- | --- | --- | --- | --- | --- | --- | --- | --- | --- |
|  | Treatment | LS Means | SEM | P-value | LS Means | SEM | P-value | LS Means | SEM | P-value | LS Means | SEM | P-value |
| IFN-α | Control | 1.70^x^ | 0.429 | 0.052 | 1.84 | 0.685 | 0.685 | 1.12 | 0.269 | 0.610 | 15.1 | 2.58 | 0.808 |
|  | FS | 2.83^y^ | 0.328 |  | 2.19 | 0.491 |  | 1.30 | 0.206 |  | 14.3 | 1.92 |  |
| IFG-γ | Control | 3.97 | 0.305 | 0.759 | 7.11 | 0.341 | 0.360 | 2.84 | 0.159 | 0.217 | 15.9 | 1.28 | 0.121 |
|  | FS | 3.86 | 0.233 |  | 6.71 | 0.260 |  | 3.09 | 0.121 |  | 18.8 | 1.20 |  |
| IL-1α | Control | 7.12 | 0.405 | 0.208 | 9.63 | 0.579 | 0.380 | 7.25 | 0.534 | 0.890 | 9.64 | 1.87 | 0.198 |
|  | FS | 7.78 | 0.309 |  | 8.97 | 0.428 |  | 7.65 | 0.471 |  | 13.0 | 1.62 |  |
| IL-1β | Control | 2.22 | 0.408 | 0.574 | 1.79 | 0.291 | 0.208 | 2.48 | 0.421 | 0.628 | 12.3 | 2.10 | 0.643 |
|  | FS | 1.93 | 0.311 |  | 1.31 | 0.223 |  | 2.75 | 0.321 |  | 13.5 | 1.66 |  |
| IL-6 | Control | 2.66 | 0.348 | 0.928 | 3.70 | 0.349 | 0.204 | 3.02 | 0.183 | 0.647 | 14.0 | 2.40 | 0.515 |
|  | FS | 2.62 | 0.278 |  | 3.14 | 0.225 |  | 2.91 | 0.140 |  | 12.0 | 1.89 |  |
| IL-8 | Control | 0.774 | 0.277 | 0.294 | 1.88 | 0.334 | 0.325 | 2.63 | 0.453 | 0.273 | 25.5 | 1.88 | 0.940 |
|  | FS | 1.14 | 0.196 |  | 1.45 | 0.255 |  | 3.28 | 0.346 |  | 25.3 | 1.53 |  |
| IL-10 | Control | 3.12 | 0.370 | 0.237 | 3.18^y^ | 0.023 | 0.097 | 1.77^a^ | 0.285 | 0.035 | 11.2 | 1.34 | 0.706 |
|  | FS | 2.55 | 0.283 |  | 3.13^x^ | 0.018 |  | 2.78^b^ | 0.247 |  | 11.9 | 1.16 |  |
| IL-12α | Control | 6.53 | 1.15 | 0.922 | 2.21 | 0.649 | 0.548 | 3.58 | 0.519 | 0.596 | 9.36 | 2.76 | 0.498 |
|  | FS | 6.38 | 0.913 |  | 1.71 | 0.479 |  | 3.94 | 0.414 |  | 11.9 | 2.39 |  |
| IL-12β | Control | 5.04 | 0.316 | 0.236 | 2.94 | 0.349 | 0.109 | 3.78^x^ | 0.263 | 0.085 | 11.7 | 2.15 | 0.575 |
|  | FS | 4.55 | 0.242 |  | 2.20 | 0.266 |  | 4.40^y^ | 0.210 |  | 13.3 | 1.87 |  |
| TNF-α | Control | 11.2 | 0.891 | 0.110 | 8.33 | 1.25 | 0.488 | 20.0 | 0.475 | 0.748 | 9.02 | 2.23 | 0.325 |
|  | FS | 13.0 | 0.681 |  | 9.42 | 0.886 |  | 20.2 | 0.363 |  | 12.1 | 1.93 |  |
| TGF-β | Control | 12.3^y^ | 0.208 | 0.050 | 10.2 | 0.228 | 0.419 | 10.7 | 0.206 | 0.356 | 10.0^b^ | 0.641 | 0.073 |
|  | FS | 11.7^x^ | 0.153 |  | 9.94 | 0.174 |  | 10.5 | 0.146 |  | 11.7^a^ | 0.566 |  |

Note: SEM = standard error of the mean. ^a–b^ Different superscripts within a row indicate a significant difference (P<0.05), ^x-y^ different superscripts within a row indicate a tendency difference (P<0.1), ^1^Dietary treatment = additional Fysal^®^ Solute (FS) at 2 kg/ton

**Table 3** - Effects of treatment on cytokine expression in jejunum, ileum and colon tissue of piglets at day 45

|  |  | Jejunum | | | Ileum | | | Colon | | | PBMC | | |
| --- | --- | --- | --- | --- | --- | --- | --- | --- | --- | --- | --- | --- | --- |
|  | Treatment | LS Means | SEM | P-value | LS Means | SEM | P-value | LS Means | SEM | P-value | LS Means | SEM | P-value |
| IFN-α | Control | 2.62 | 0.501 | 0.581 | 3.26 | 0.610 | 0.871 | 2.30 | 0.328 | 0.670 | 16.6^y^ | 1.84 | 0.772 |
|  | FS | 2.98 | 0.409 |  | 3.12 | 0.528 |  | 2.48 | 0.267 |  | 15.9^y^ | 1.40 |  |
| IFG-γ | Control | 3.82^a^ | 0.348 | 0.017 | 6.86 | 0.301 | 0.261 | 3.03 | 0.106 | 0.065 | 17.0 | 1.70 | 0.404 |
|  | FS | 2.63^b^ | 0.302 |  | 6.40 | 0.261 |  | 2.76 | 0.087 |  | 15.2 | 1.30 |  |
| IL-1α | Control | 4.26^a^ | 0.348 | 0.017 | 6.86 | 0.301 | 0.261 | 3.63 | 0.435 | 0.124 | 16.9 | 2.30 | 0.233 |
|  | FS | 3.06^b^ | 0.302 |  | 6.40 | 0.261 |  | 2.59 | 0.332 |  | 13.1 | 1.96 |  |
| IL-1β | Control | 5.55 | 0.509 | 0.452 | 5.44 | 0.632 | 0.873 | 4.26 | 0.381 | 0.178 | 16.0^a^ | 1.30 | 0.021 |
|  | FS | 5.05 | 0.389 |  | 5.31 | 0.516 |  | 3.57 | 0.311 |  | 11.6^b^ | 1.17 |  |
| IL-6 | Control | 2.63 | 0.309 | 0.221 | 2.73 | 0.340 | 0.230 | 3.11 | 0.316 | 0.542 | 17.9 | 2.20 | 0.948 |
|  | FS | 2.10 | 0.279 |  | 3.29 | 0.295 |  | 3.37 | 0.258 |  | 17.7 | 1.75 |  |
| IL-8 | Control | 2.34 | 0.286 | 0.395 | 2.41^b^ | 0.250 | 0.034 | 4.16 | 0.326 | 0.252 | 24.2 | 2.17 | 0.331 |
|  | FS | 2.03 | 0.219 |  | 3.15^a^ | 0.204 |  | 3.64 | 0.295 |  | 26.9 | 1.60 |  |
| IL-10 | Control | 2.52^b^ | 0.274 | 0.035 | 3.10 | 0.013 | 0.378 | 2.74^b^ | 0.225 | 0.007 | 12.8 | 1.65 | 0.654 |
|  | FS | 1.70^b^ | 0.237 |  | 3.12 | 0.011 |  | 1.74^a^ | 0.195 |  | 11.8 | 1.26 |  |
| IL-12α | Control | 8.31 | 0.983 | 0.252 | 2.08 | 0.861 | 0.822 | 3.62 | 0.626 | 0.351 | 17.3 | 2.84 | 0.390 |
|  | FS | 6.84 | 0.751 |  | 2.33 | 0.703 |  | 4.41 | 0.524 |  | 14.1 | 2.32 |  |
| IL-12β | Control | 4.73 | 0.344 | 0.417 | 2.24 | 0.209 | 0.401 | 4.58^x^ | 0.238 | 0.070 | 17.0 | 2.85 | 0.250 |
|  | FS | 4.35 | 0.298 |  | 2.47 | 0.181 |  | 4.00^y^ | 0.182 |  | 12.6 | 2.43 |  |
| TNF-α | Control | 10.1 | 1.11 | 0.360 | 8.79 | 1.15 | 0.512 | 20.3 | 1.12 | 0.561 | 16.2 | 2.89 | 0.294 |
|  | FS | 8.73 | 0.903 |  | 9.80 | 0.993 |  | 19.4 | 0.915 |  | 12.1 | 2.47 |  |
| TGF-β | Control | 12.34 | 0.170 | 0.256 | 10.3 | 0.149 | 0.598 | 11.2 | 0.199 | 0.146 | 14.1 | 1.23 | 0.451 |
|  | FS | 12.07 | 0.154 |  | 10.2 | 0.129 |  | 10.8 | 0.172 |  | 12.9 | 1.00 |  |

Note: SEM = standard error of the mean. ^a–b^ Different superscripts within a row indicate a significant difference (P<0.05), ^x-y^ different superscripts within a row indicate a tendency difference (P<0.1), ^1^Dietary treatment = additional Fysal^®^ Solute (FS) at 2 kg/ton
